# “Comet tail sign”: A pitfall of post-gadolinium magnetic resonance imaging findings for metastatic brain tumors

**DOI:** 10.1007/s11060-016-2069-1

**Published:** 2016-02-02

**Authors:** Koichi Mitsuya, Yoko Nakasu, Yoshitaka Narita, Satoshi Nakasu, Makoto Ohno, Yasuji Miyakita, Masato Abe, Ichiro Ito, Nakamasa Hayashi, Masahiro Endo

**Affiliations:** Division of Neurosurgery, Shizuoka Cancer Center, 1007 Naga-izumi, Shizuoka, 411-8777 Japan; Division of Pathology, Shizuoka Cancer Center, Shizuoka, Japan; Division of Diagnostic Radiology, Shizuoka Cancer Center, Shizuoka, Japan; Division of Neurosurgery, National Cancer Center, Tokyo, Japan; Division of Neuro-oncology, Kusatsu General Hospital, Shiga, Japan

**Keywords:** Atypical enhancement pattern, Extravasation, Gadolinium, Magnetic resonance imaging, Metastatic brain tumor

## Abstract

A highly enhanced cap attached to the surface of metastatic tumors in the brain parenchyma is occasionally encountered on magnetic resonance (MR) images. This atypical enhanced cap tends to occur in severe peritumoral edema and may produce the characteristic bulge of a metastatic mass lesion termed the “comet tail sign” (CTS). The purpose of this study was to demonstrate the features of the CTS using MR imaging and pathological findings, and to clarify its clinical relevance. We selected 21 consecutive cases of newly diagnosed metastases from MR imaging studies that demonstrated the CTS; all had diffuse peritumoral edema. The MR T2-weighted images showed similarly homogenous and high intensity signals in both the tail and peritumoral edema. Fourteen of the 21 patients underwent surgical resection of their tumors, and 12 tails were separately removed for pathological examination, no tumor cells which revealed. We speculate that the CTS does not contain neoplastic tissues but is observed as a result of the leakage of contrast medium from the tumor body into the interstitial space of the white matter. Although CTS is a peculiar and uncommon enhancement pattern, it has clinical significance in determining the extent of the margin for invasive local treatments, such as surgical resection or stereotactic radiotherapy; this is particularly true in and near the eloquent areas.

## Introduction

Metastatic brain tumors occur in 8.5–40 % of patients with systemic cancers [1, 2]. Recent advances in the systemic management of cancer patients and novel imaging techniques have led to an increase in the number of long-term survivors with brain metastases [3]. Magnetic resonance (MR) imaging is the most reliable diagnostic technique for metastatic brain tumors [4, 5]. Typical MR images show tumor mass lesions of well-circumscribed enhancement; they have isointense or high-intensity signals on T2-weighted images and low-intensity signals on T1-weighted images [6, 7]. These lesions are surrounded by areas of peritumoral edema of various sizes [8].

We found a highly enhanced peripheral area that was attached to an enhanced main tumor mass in the brain parenchyma. This peripheral enhancement had a constant pattern of intense and homogenous signals. It appeared to be budding-off from the main tumor mass, which we recognized as a unique feature among brain metastases. A series of cases with this characteristic enhancing pattern were compiled. We termed this pattern the “comet tail sign” (CTS) because of its resemblance to a shooting star with a tail.

The purpose of the current study was to retrospectively demonstrate the MR features of CTS and to clarify its clinical relevance in our patient series.

### Patients and methods

This study involved a retrospective review of 21 patients who exhibited the characteristic caps of intense peripheral enhancement that were attached to the surface of metastatic brain tumors. MR imaging was performed using a 1.5T MR imaging system (Intera: Philips Healthcare, Best, The Netherlands). From all of the 21 patients, unenhanced spin-echo T1-weighted axial images, fast spin-echo T2-weighted axial images, and contrast-enhanced 3D-gradient echo T1WIs were obtained. The MR imaging parameters used were as follows: repetition time (TR)/echo time (TE), 350–600/12 ms; number of excitations (NEX), 1; matrix, 320 × 225 for T1WI and TR/TE of 4500/90 ms; flip angle, 15°; NEX, 1, matrix, 256 × 205 for T2WI. The other parameters were: 5-mm section thickness; 0.5-mm gap; and 230-mm field of view (FOV). For contrast-enhanced T1W images, we routinely commenced reference imaging at 1 min after a single intravenous injection of gadopentetate dimeglumine (Magnevist: Bayer Health Care Pharmaceuticals, Wayne, NJ, USA) at a dose of 0.2 mL/kg. It took approximately 40 s to complete the reference imaging, and we proceeded to sagittal, coronal, and axial scans in due order. It took approximately 2 min to obtain images of each plane. Consequently, our routine contrast imaging started 2 min after the injection of contrast material, and finished after an interval of approximately 6 min. The reported cases were consistently examined using this protocol. The parameters for contrast-enhanced T1WI were: TR/TE, 14/4.6 ms; flip angle, 15°; NEX, 1; matrix, 256 × 179; section thickness, 4 mm; gap, 2 mm; and FOV, 230 mm.

All of the 21 patients had characteristic comet-shaped main masses and tails in the brain on post-contrast MR images. All showed increased T2WI signals from the tails in diffuse perifocal edema. Fourteen patients underwent surgical removal of the masses, and 12 of them underwent biopsies or resection of the tails. Two representative cases are described below with pathological confirmation regarding the tails.

## Results

Twenty-one patients who had metastatic brain tumors with CTS were observed at our hospital from August 2008 to March 2015 (Table 1). All of these patients presented with newly diagnosed metastases in the brain. Median patient age was 66 (range, 44–81) years with a female-to-male ratio of 3:18. Monitoring of samples in one of the institutes indicated that the incidence of CTS was 3 % (10 patients out of 329 with newly diagnosed metastatic brain tumors over 1 year).

**Table 1:** Clinical and pathological characteristics of patients with comet tail sign

Case	Age	Sex	Primary	Pathology	Location	Tumor volume (ml)	Edema volume (ml)	Edema index	Comet tail MR; T2 intensity	Treatment	Comet tail; pathology	Remnant tail; post Op	Remnant tail; follow-up	Local control
1	72	M	Lung	ad	Occipital	20	285.7	14.3	High	Op + RT	No tumor cell, MVP	(+)	Disapeared	No rec
2	57	M	Lung	pleo	Parietal	23	200.2	8.7	High	Op + RT	No tumor cell, MVP	(+)	Disapeared	No rec
3	64	M	Lung	large	Frontal	7.5	146.9	19.6	High	Op + RT	No tumor cell, MVP	None	None	No rec
4	60	M	Colon	ad	Occipital	35.5	142.1	4	High-iso	Op + RT	No tumor cell, MVP	(+)	Disapeared	No rec
5	61	M	Pharyngs	sq	Parietal	7.1	85.7	12.1	High	Op + RT	No tumor cell, MVP	(+)	Disapeared	No rec
6	79	M	Esophagus	sq	Occipital	29.8	175.9	5.9	High	Op + RT	No tumor cell, MVP	None	None	No rec
7	81	M	Lung	ad	Temporal	41.5	265	6.4	High	Op + RT	No tumor cell, MVP	none	None	No rec
8	77	F	Uterus body	cs	Frontal	50	226.8	4.5	High	Op + RT	No tumor cell, MVP	(+)	Disapeared	No rec
9	70	M	Gastric	ad	Cerebellar	57.9	83.7	1.4	High	Op + RT	No tumor cell, MVP	None	None	No rec
10	60	M	Lung	ad	Frontal	47.5	185.5	3.9	High	Op + RT	No tumor cell, MVP	(+)	Disapeared	No rec
11	61	F	Breast	ad	Frontal	29.7	373	12.6	High	Op	No tumor cell, MVP	(+)	Disapeared	no rec
12	60	M	Gastric	ad	Frontal	15.6	219.2	14.1	High	Op	No tumor cell, MVP	None	None	No rec
13	62	M	Lung	ad	Cerebellar	12.8	99.6	7.8	High	Op + SRS	N.A.	(+)	Disapeared	No rec
14	76	M	Colon	ad	Occipital	22.2	159.3	7.2	High-iso	Op	N.A.	(+)	Disapeared	Main mass rec
15	78	M	Gastric	ad	Frontal	4.1	150.2	37.5	High	SRT	/	/	/	No rec
16	70	M	Lung	sq	Occipital	5.4	117.5	23.5	High	SRT	/	/	/	Main mass rec
17	54	M	Lung	ad	Temporal	3.8	110.5	29.1	High	SRT	/	/	/	Radiation necrosis
18	78	M	Colon	sd	Frontal	3.4	84	24.7	High	SRT	/	/	/	No rec
19	44	M	Lung	ad	Parietal	17.1	107.7	6.3	High	WBRT	/	/	/	No rec
20	64	M	Lung	small	Cerebellar	15.6	78.4	5	High	WBRT	/	/	/	No rec
21	63	F	Lung	ad	Temporal	2.4	85.8	40	High	WBRT	/	/	/	No rec

*ad* adenocarcinoma, *pleo* pleomorphic carcinoma, *large* large-cell carcinoma, *small* small-cell carcinoma, *sq* squamous-cell carcinoma, *cs* carcinosarcoma, *rec* recurrence, *MVP microvascular proliferation, N.A*. not analyzed, *OP* operation, *RT* Radiotherapy, *SRS* stereotactic radiosurgery,*SRT* stereotactic radiotherapy*, WBRT* whole brain radiotherapy

A majority of the patients presented with lung cancer (11 cases [52 %]); seven had adenocarcinoma, and one each had squamous cell carcinoma, small-cell carcinoma, large-cell carcinoma, and pleomorphic carcinoma. The remaining patients had gastric cancer (3 cases) and colorectal cancer (3 cases); in addition, there was one case each of breast, pharyngeal, esophageal and uterus body cancer.

**Fig. 1:**
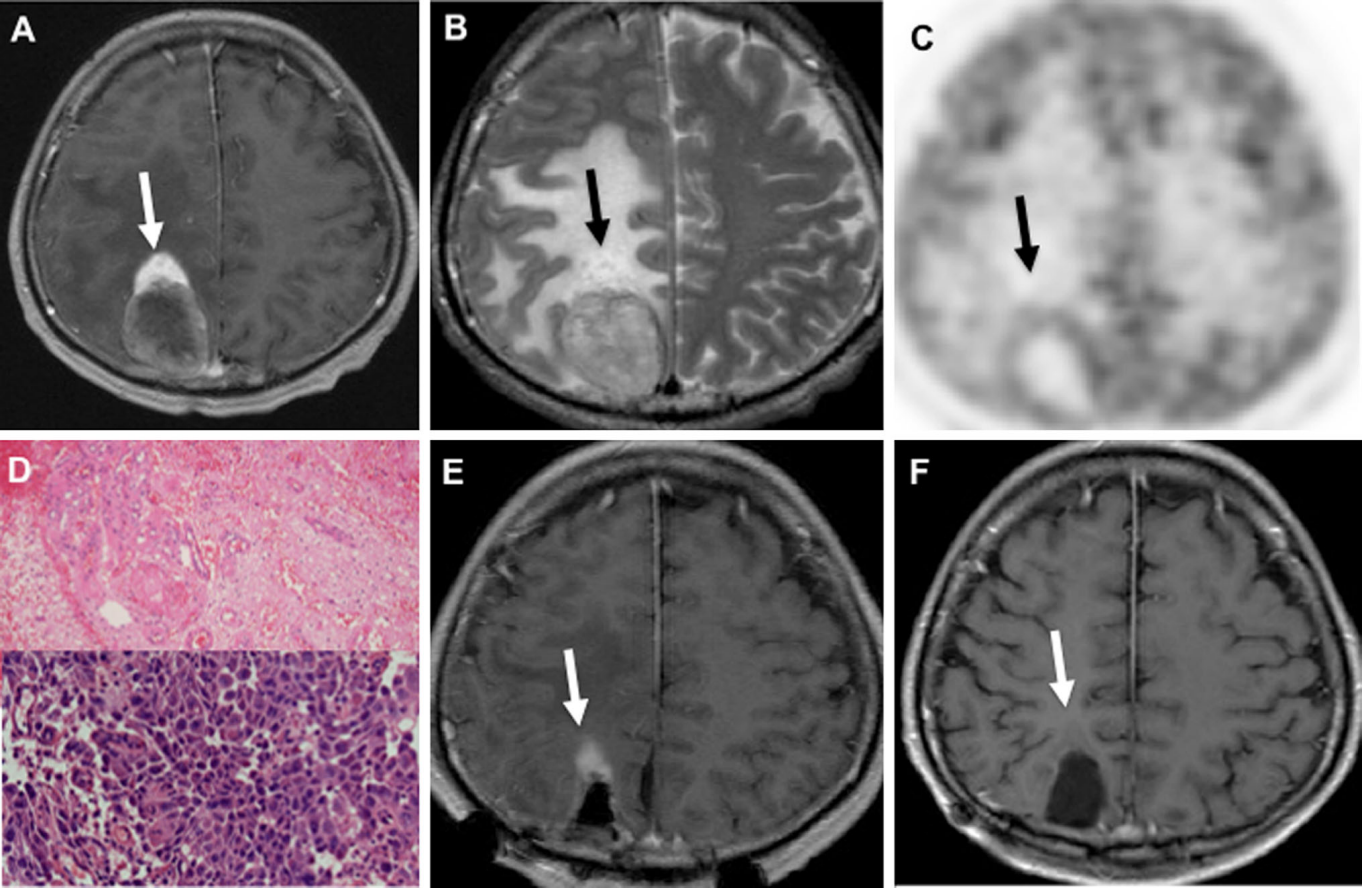
**a** T1-contrast enhancement (CE) image demonstrating the comet tail sign with characteristic perilesional intense contrast enhancement (*white arrow*) in the *right* parietal region. **b** T2-weighted images demonstrating diffuse brain edema. The tail of the comet has a high-intensity signal equal to that of the perifocal edema (*black arrow*). The main body is surrounded by sulci, and the tail is located at the neck of the gyrus. **c** 2-Deoxy-2[F-18]fluoro-d-glucose-positron emission tomography image showing no accumulation of tracer in the tail of the comet tail sign (CTS). **d** Photomicrographs of stained tissue sections showing a pleomorphic carcinoma in the main mass (*lower column*), but no neoplastic cells in the tail region (*upper column*) (hematoxylin and eosin stain). **e** The tumor and a part of the tail were resected separately. Postoperative MR image showing the residual tail of the CTS (postoperative day 2). **f** This residual tail of CTS had disappeared on follow-up MR images at 3 months after surgery

**Fig. 2:**
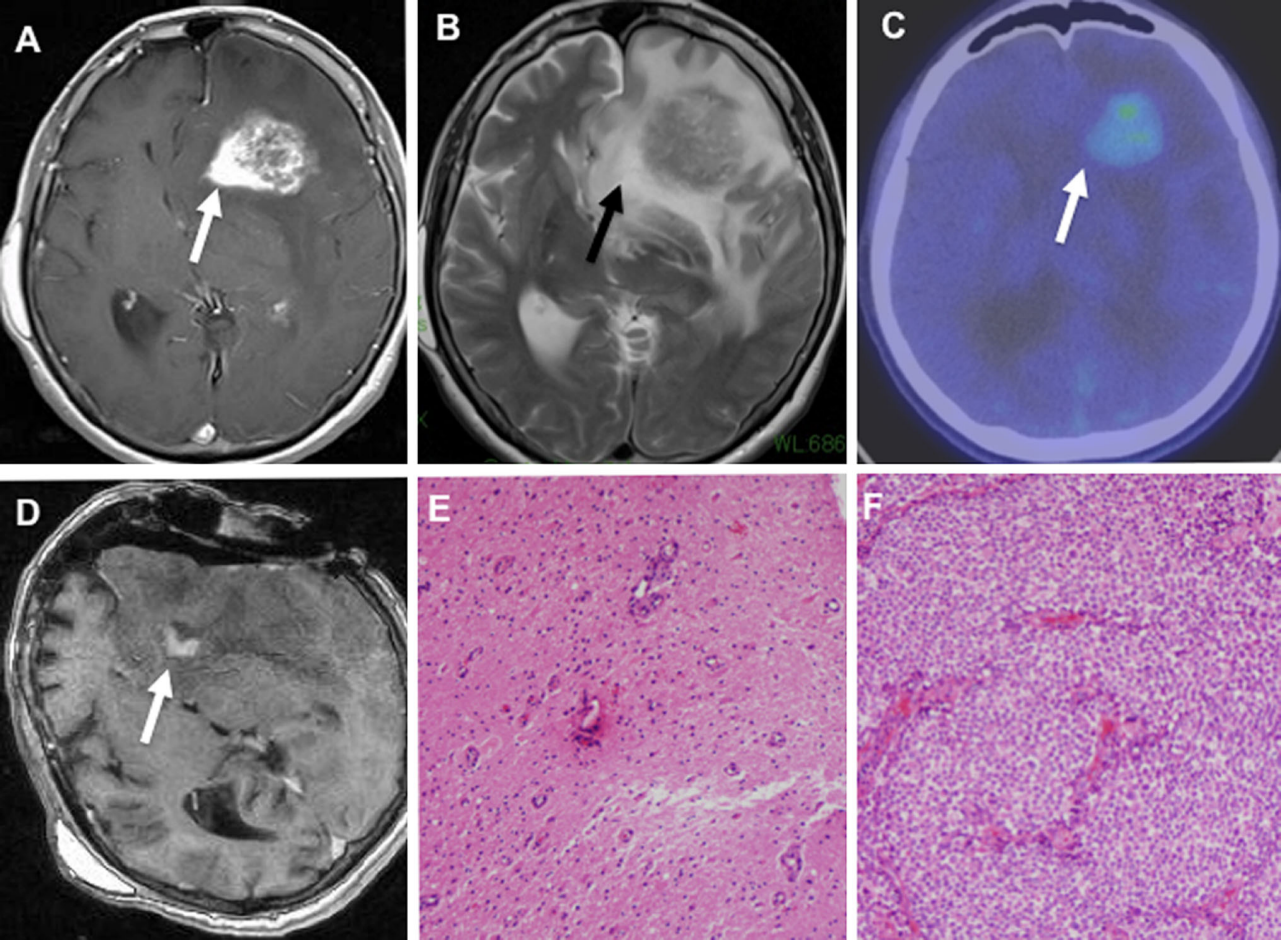
**a** T1-CE image showing a *left* frontal mass with a CTS (*white arrow*). **b** T2-weighted image demonstrating severely diffuse brain edema. The tail of the comet has a high-intensity signal equivalent to the perifocal edema (*white* **a** and *black* **b** *arrows*). The tail of the comet protrudes into the anterior horn of the lateral ventricle. **c** Methionine-positron emission tomography images showing no accumulation of tracer in the tail of the CTS. **d** Intraoperative T1-CE showing that the main body was removed. The remnant of the tail was removed after imaging. **e** Photomicrograph showing microvascular proliferation, but no neoplastic cells in the CTS (hematoxylin and eosin stain). **f** Photomicrograph showing an adenocarcinoma (*breast cancer*) in the main lesion

**Fig. 3:**
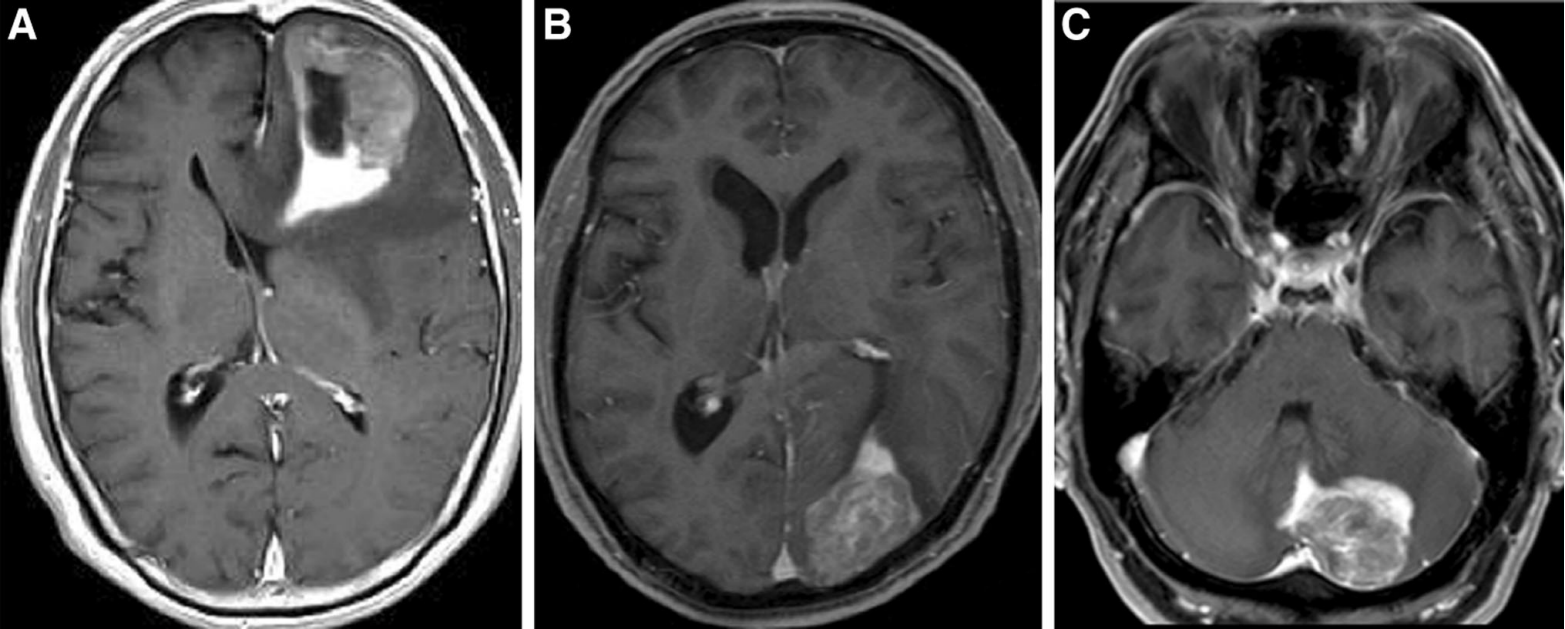
T1-CE images demonstrating masses with the CTS in three different patients. The tails protrude into the anterior (**a**) and posterior (**b**) horns of the lateral ventricle, and into the forth ventricle (**c**)

**Fig. 4:**
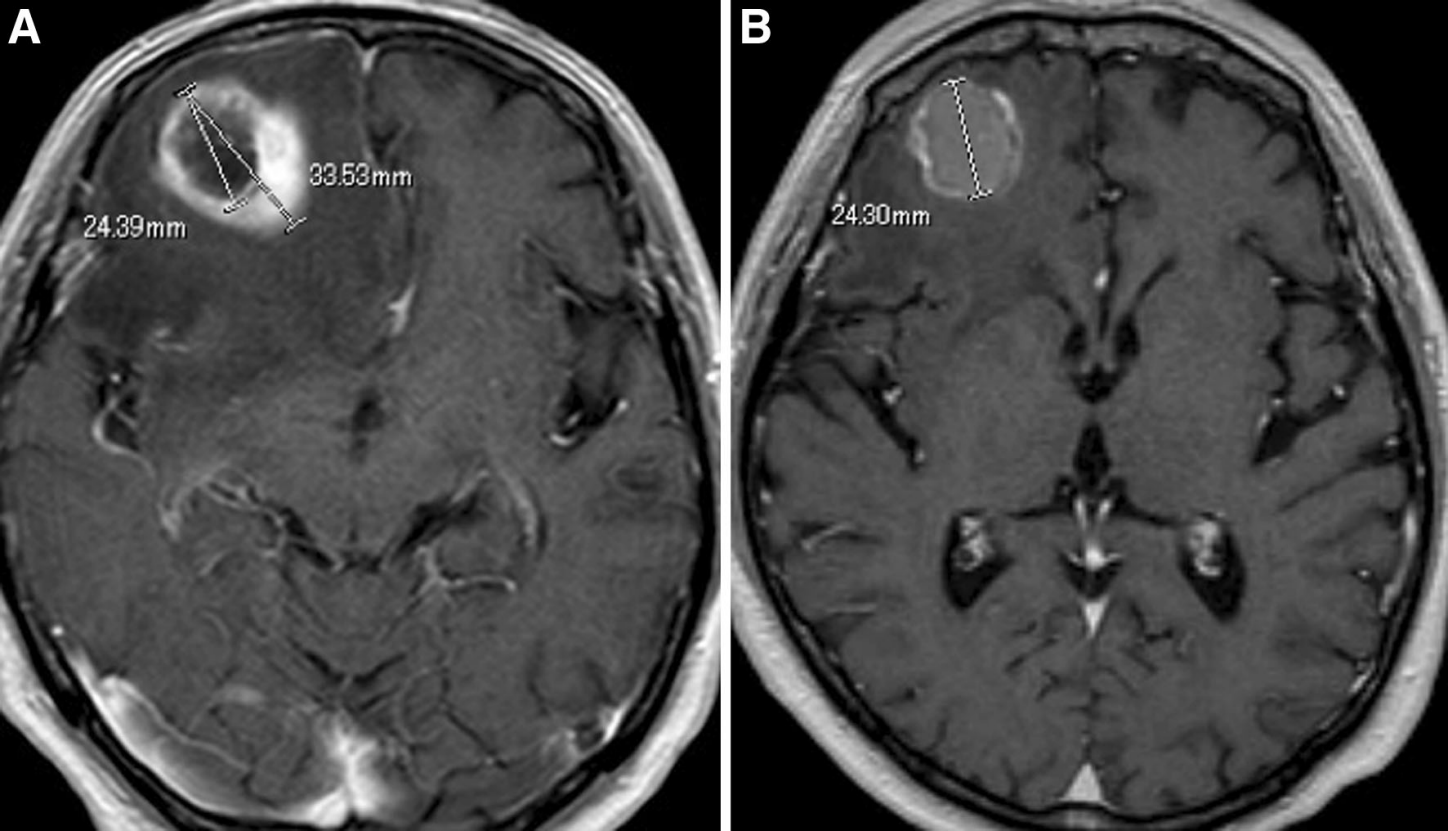
**a** MR T1-CE showing the CTS in the *right* frontal metastatic brain tumor of a patient with lung cancer. **b** MR T1-CE image showing that the tail of the CTS had disappeared after administration of corticosteroids

Post-gadolinium T1WI demonstrated more intense and homogenous enhancement in the tail than in the main body (Fig. 3). T2WI revealed that the main body of the tumor had a signal that ranged from low-intensity to isointensity; however, the tail of the CTS had a high-intensity signal buried in peritumoral diffuse edema (Figs. 1b and 2b). When these metastatic brain tumors with CTS were situated near the ventricle, the tails always pointed towards the ventricle; more specifically, they pointed towards the anterior horn (Fig. 3a) and the posterior horn (Fig. 3b) of the lateral ventricle, and towards the fourth ventricle (Fig. 3c). The tail of the CTS disappeared concurrently with the decrease in perifocal edema in a single patient after administration of dexamethasone for 1 week (Fig. 4).

Fourteen patients underwent surgical resection, among which 12 involved both the main masses and the tails. Pathological examination of the tails of the CTS revealed edema and micro-vascular proliferation, but no neoplastic cells (Figs. 1d and 2e). Nine patients had remnant comet tails on MR images at 2 days after surgery (Table 1). Considered overall, follow-up MR images demonstrated the disappearance of the tails regardless of whether patients had received adjuvant therapies a few months after surgery.

Among the seven patients who did not undergo surgical resection, four underwent stereotactic radiotherapy targeted exclusively at the main masses. The tails of the CTS disappeared and never recurred after stereotactic radiotherapy. They also disappeared and did not recur in the other three patients who underwent whole brain radiation therapy (Table 1).

### Case 1

A 57-year-old man presented with a week-long history of left hemiparesis and left spatial agnosia. MR images revealed a single brain tumor that was 45 mm in diameter, with a CTS and diffuse brain edema in the right parietal lobe (Fig. 1a, b). Systemic examination revealed lung cancer. Because the tail showed a high-intensity signal on T2WI (Fig. 1a, b) similar to the peritumoral edema, we suspected that it consisted of tissues that differed from the main body of the tumor. Using 2-deoxy-2[F-18]fluoro -d-glucose positron emission tomography, it was demonstrated that there was no accumulation of tracer in the tail of the CTS (Fig. 1c). The main mass and a part of the tail were resected separately. Pathological examination revealed a pleomorphic carcinoma in the main mass (Fig. 1d lower column), but no neoplastic cells in the tail region (Fig. 1d upper column). Postoperative MR images demonstrated a residual tail of the CTS (postoperative day 2; Fig. 1e), but this had disappeared on the follow-up MR image at 3 months after surgery (Fig. 1f).

### Case 2

A 61-year-old woman presented with urinary incontinence. She had undergone breast cancer surgery 15 years previously, followed by six courses of postoperative chemotherapy, and tamoxifen therapy for 2 years. MR images revealed a large single mass of 43 mm in diameter in the left frontal lobe. The tail of the CTS extended towards the anterior horn of the lateral ventricle (Fig. 2a) in diffuse edema (Fig. 2b).

Methionine-positron emission tomography images demonstrated no accumulation of tracer in the tail of the CTS (Fig. 2c). After complete removal of the tumor, the residual tail of the CTS was resected separately under the guidance of intraoperative MR imaging (Fig. 2d). Pathological examination revealed an adenocarcinoma in the main mass (Fig. 2f), and microvascular proliferation in the tail region of the CTS, but no neoplastic cells in the white matter gliosis (Fig. 2e).

## Discussion

In the present study, we present the findings from 21 patients with newly diagnosed metastatic brain tumors who had a peculiar enhancement feature on MR images termed the CTS. The CTS showed exhibited specific features on T2-WI and post-gadolinium T1WI. Although the presence of a CTS is not a common finding for metastatic brain tumors, it is a reliable marker; it can be a serious pitfall when the margins of metastatic tumors are delineated, as revealed by pathological examination in our study.

The CTS consists of a main lesion and a tail. The common features of CTS observed in this study can be summarized as follows. First, it had the appearance of an enhanced mass consisting of two components attached to each other. The tail exhibited more intense and homogenous enhancement than the main lesion on post-gadolinium T1WI. Second, the tail had the same signal intensity as the peritumoral edema; however, the main lesion showed isointensity or low-signal intensity on T2WI as is generally found. Third, the tail always extended towards the deep white matter and ventricles. These findings suggest that the CTS is composed of tissue that differs from the main neoplastic lesion.

In the present study, all cases exhibited severe peritumoral edema on T2WI. They had higher edema indexes than those previously reported for brain metastases, with a median value of 8.7 (range, 1.4–40.0; 95 % confidence interval [CI], 8.6–18.9) in our CTS cases, while the median value was 3.3 (range, 1.0–9.1; 95 % CI 2.8–5.0) in previously reported cases of brain metastases [8]. This severe edema may be related to one of the causes of the CTS. Our hypothesis is that chemical substances that were extravasated from the tumor body could have been retained and concentrated in the local white matter; this occurred when the white matter was anatomically surrounded by sulci or the brain surface, and connected only towards the deep white matter and the periventricular region [9]. These chemical substances may include vasodilators and contrast medium. In the current study, the tail of the CTS disappeared along with perifocal edema after the administration of sufficient corticosteroids (Fig. 4). Considered overall, we suggest that the CTS may represent extravasation of contrast medium from the blood-tumor barrier and its retention within the interstitial space of white matter outside the tumor [10, 11].

The CTS is of clinical importance regarding the local treatment of brain metastasis, particularly in the eloquent areas, although it is an uncommon finding. In all of the 12 patients who underwent biopsy or resection of the tails, pathological examination did not reveal tumor cells or microvascular proliferation in the edematous gliotic white matter. For surgical resection, total removal of the CTS is not required, although biopsy may be needed for confirmation of non-neoplastic conditions. Regarding the planning of radiosurgery, the CTS poses a challenge in relation to tumor delineation. Its presence may also indicate an increased risk of late radiation effects in the brain as a result of excessive radiation dose, or of poor tumor control as a result of the delivery of an insufficient radiation dose to the tail region.

Brain metastasis accompanied by CTS may possess different biological features to those of typical brain metastasis. All of the main lesions in our series were situated in the superficial subcortical layers and were attached to tails pointing towards the deep or periventricular white matter. Pathological examination revealed glomeruloid microvascular proliferation in the tails of the CTS in several cases, as previously reported [12, 13]. This hypervascularity may be another possible cause of the CTS.

A limitation of the present study was its retrospective nature. Of the 21 patients who exhibited the CTS detected during routine MR examination for brain metastasis, only 12 underwent pathological confirmation concerning the tail region. The mechanism behind the formation of the attached tail may include compound phenomena. The natural history, surgical and radiologic-pathologic correlation, and treatment outcomes should be analyzed in a larger cohort of patients.

## Conclusion

We reported on the MR features of an atypical perilesional contrast enhancement in metastatic brain tumors termed the “comet tail sign” and abbreviated as CTS. The CTS may represent a pitfall in the assessment of metastatic brain tumors that demonstrate enhancement of potentially non-neoplastic tissue. Although it is an uncommon MR finding, the CTS has clinical significance in treatment planning regarding the extent of invasive local treatment, such as surgical resection or stereotactic radiotherapy.
